# Cleavage of DNA Substrate Containing Nucleotide Mismatch in the Complementary Region to sgRNA by Cas9 Endonuclease: Thermodynamic and Structural Features

**DOI:** 10.3390/ijms251910862

**Published:** 2024-10-09

**Authors:** Svetlana V. Baranova, Polina V. Zhdanova, Anastasia D. Koveshnikova, Pavel E. Pestryakov, Ivan P. Vokhtantsev, Alexander A. Chernonosov, Vladimir V. Koval

**Affiliations:** 1 Institute of Chemical Biology and Fundamental Medicine, Siberian Branch of the Russian Academy of Sciences (SB RAS), 630090 Novosibirsk, Russia; polina.zhdanova@niboch.nsc.ru (P.V.Z.); a.koveshnikova@g.nsu.ru (A.D.K.); pestryakov@niboch.nsc.ru (P.E.P.); ivanvohtancev@gmail.com (I.P.V.); alexander.chernonosov@niboch.nsc.ru (A.A.C.); 2 Department of Natural Sciences, Novosibirsk State University, 630090 Novosibirsk, Russia

**Keywords:** CRISPR/Cas systems, Cas9 activity, sgRNA, oligonucleotide mismatch, cleavage, thermodynamics, molecular dynamic simulations

## Abstract

The non-ideal accuracy and insufficient selectivity of CRISPR/Cas9 systems is a serious problem for their use as a genome editing tool. It is important to select the target sequence correctly so that the CRISPR/Cas9 system does not cut similar sequences. This requires an understanding of how and why mismatches in the target sequence can affect the efficiency of the Cas9/sgRNA complex. In this work, we studied the catalytic activity of the Cas9 enzyme to cleave DNA substrates containing nucleotide mismatch at different positions relative to the PAM in the “seed” sequence. We show that mismatches in the complementarity of the sgRNA/DNA duplex at different positions relative to the protospacer adjacent motif (PAM) sequence tend to decrease the cleavage efficiency and increase the half-maximal reaction time. However, for two mismatches at positions 11 and 20 relative to the PAM, an increase in cleavage efficiency was observed, both with and without an increase in half-reaction time. Thermodynamic parameters were obtained from molecular dynamics results, which showed that mismatches at positions 8, 11, and 20 relative to the PAM thermodynamically stabilize the formed complex, and a mismatch at position 2 of the PAM fragment exerts the greatest stabilization compared to the original DNA sequence. The weak correlation of the thermodynamic binding parameters of the components of the Cas9/sgRNA:dsDNA complex with the cleavage data of DNA substrates containing mismatches indicates that the efficiency of Cas9 operation is mainly affected by the conformational changes in Cas9 and the mutual arrangement of sgRNA and substrates.

## 1. Introduction

Bacteria invent CRISPRs as an adaptive immune system to protect themselves against viruses and cut viral DNA. Today, the CRISPR/Cas system has become one of the key tools for genome editing and offers a promising technology for use in the field of personalized medicine [1,2,3,4,5]. Promising applications for this technology include the treatment of cancers, cardiovascular diseases, sickle cell anemia, and neurodegenerative disease. There are several different types of infectious diseases that can potentially be treated with CRISPR, including viruses, bacteria, and fungi.

The widely used bipartite system CRISPR/Cas9, which includes only the Cas9 enzyme and a single-stranded guide RNA (sgRNA), targets the entire complex to specific genomic regions [1,2]. The principles of Cas9/sgRNA complex assembly as well as the location of the guide RNA before target DNA interaction are best described by Jiang et al. [3]. Structural and biochemical data show that the binding of sgRNA triggers structural rearrangement in Cas9 from an inactive conformation to one able to recognize a specific DNA sequence [3]. A specific protospacer adjacent motif (PAM) near a specific sequence in the DNA is also required for Cas9 to function. The recognition of target DNA by Cas9/sgRNA is mediated by three-dimensional collision, and a failure to find the PAM results in rapid dissociation from the DNA [4,5]. In the presence of a PAM in the target DNA, the endonuclease is able to cleave both the plasmid and short linear DNA, with recognition and catalysis (cleavage) times depending on the complementarity between sgRNA and DNA [6,7].

Although Cas9 is site-specific, the CRISPR/Cas9 system still allows up to six mismatches within the complementary sgRNA sequence (Jinek et al. [1]). For example, many off-target changes were observed in human cells in the first attempts at genome editing [2,8]. It was found that Cas9 endonuclease can cleave double-stranded target DNA both in the absence of PAM and in the case of a single mismatch in the PAM [9,10]. Cas9 activity is maintained even in the case of a “wrong” substrate. Common errors include insertions, deletions, and mismatches in the target sequence [11,12]. It was recently suggested that the CRISPR/Cas9 genome and epigenome editing tool has the potential to revolutionize the field of epigenetics [13].

The efficiency of the Cas9 system is not only influenced by the presence of PAM and the specific sequence to the sgRNA, but also, with the high probability, by the correct folding of the target DNA duplex and its stability [10,12]. It was found that a mismatch in the “seed” sequence significantly reduces the rate of the DNA cleavage reaction of the target DNA. The comparison of the efficiency of the Cas9/sgRNA complex to the thermodynamic parameters of the formation of nucleic acid duplexes containing non-complementary pairs at different positions has shown that the action of Cas9 is a kinetic controlled process [12].

Although numerous studies of the Cas9 endonuclease [1,7,9,10,12,14] have elucidated the process of guide RNA binding to the target and the recognition of the PAM region, and have shown that the enzyme undergoes significant conformational rearrangements, the exact mechanisms of target DNA recognition and binding remain to be clarified. Therefore, studying the mechanisms by which the CRISPR/Cas9 system recognizes and cleaves target DNA sequences, especially in the case of mistargeting, is very important to improve the efficiency of the system. The aim of this study was to determine the recognition and cleavage rate of Cas9 to fully model complementary DNA substrates, forming a mismatched complex upon binding to sgRNA. Recent works have shown that Cas9 is much less tolerant of guide-DNA mismatches in the PAM-proximal seeding region than those in PAM-distal non-seed regions. The shift-PAM targeting expands options when designing sgRNAs and will be useful when applied in various genome-editing contexts [15,16].

## 2. Results and Discussion

Although the specificity of Cas9 function is primarily important for genomic editing of extended DNA [17], Cas9 efficiency is easier to study using model substrates up to 55–60 bp in length [18,19,20,21]. Of particular interest are the targets where the mismatch is formed during the assembly of the sgRNA:DNA complex, simulating the faulty work of Cas9 during genomic editing.

For the targeting of Cas9 to a definite sequence in the genome, the definite sequence of the sgRNA is used, which could affect Cas9 specificity by itself [8,22,23]. As a result, the tolerance of mismatches at each position strongly depends on the sgRNA sequence. One of possible reasons for this could be the different thermodynamic stability of the sgRNA:DNA duplex [22]. Therefore, when studying the influence of the mismatch position on the specificity of Cas9, it is important to consider the stability of the sgRNA:DNA complex.

In this work, 55 bp DNA duplexes with substitutions to A-T and G-C pair were designed so that the effect of the substitution on the duplex structure was minimal, but there was a mismatch in the formation of sgRNA:DNA complexes (Figure S1). Ten dsDNA substrates with a substitution to an A-T pair were designed with substitutions at different positions relative to the PAM region so that the substitutions did not lead to hairpin formation and a change in the melting temperature of the duplexes. The substrates were labeled S0, S2, S4, S5, S6, S8, S11, S13, S16, S18, and S20 (Table 1), where the number represents the position of the inverted A-T pair relative to the PAM (-GGT-). Upon interaction with the Cas9/sgRNA complex, a complex containing an A:dA (except S11 with U:dT, and S8 and S16 with G:dA) mismatch between the substrate and sgRNA was formed, which might affect the efficiency of substrate cleavage. To confirm the results obtained for substrates with the lowest (S8) and highest (S11 and S20) cleavage, the substrates with substitutions of a pair of G-Cs in similar positions were also studied (Table 1, substrates S8gc, S11gc, and S20gc). For these substrates, the mismatches in the complex between the substrate and sgRNA were G:dG for S8gc, U:dG for S11gc, and A:dG for S20gc. For all these substrates, the efficiency of the Cas9 cleavage reaction was investigated. For the most interesting results, thermodynamic parameters of duplex formation were calculated and computer modeling was performed to explain the results obtained and to suggest why the Cas9/sgRNA complex recognizes the DNA with mismatch.

### 2.1. Efficiency of Cleavage of Substrates with Mismatches

**Figure 1:**
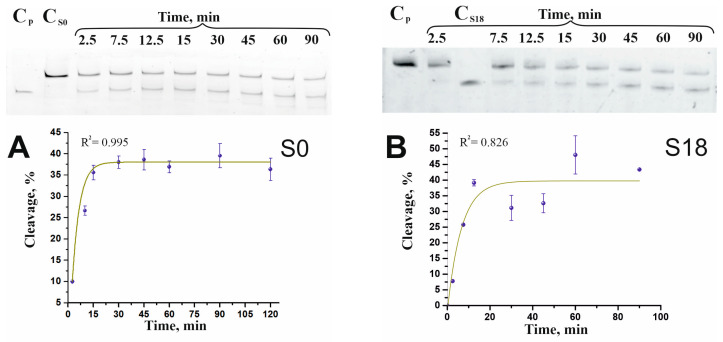

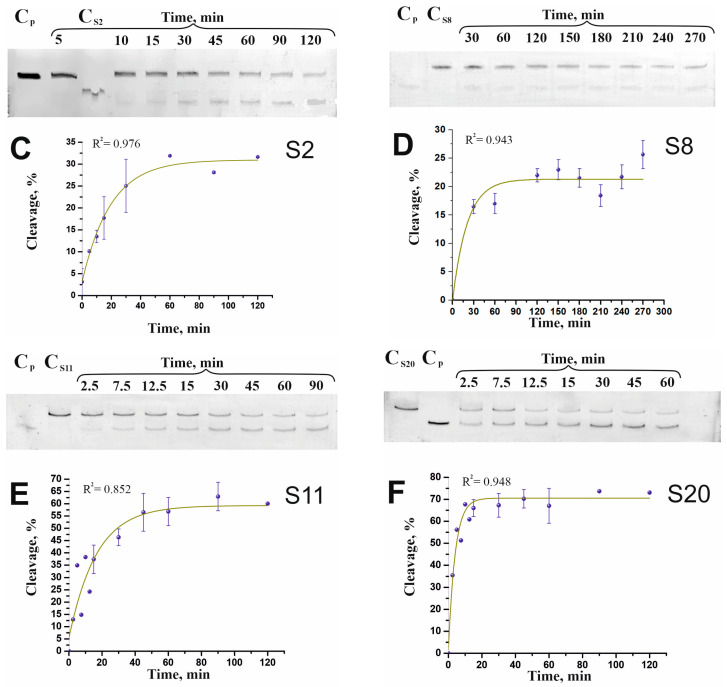
Cleavage assay of 55 bp substrate S0 (**A**), S18 (**B**), S2 (**C**), S8 (**D**), S11 (**E**), and S20 (**F**) analyzed by denaturing 15% polyacrylamide gel electrophoresis, and representation of time dependence of cleavage on graphs. Cleavage was performed using 2 nM FAM-labeled dsDNA and 50 nM complex Cas9/sgRNA (ratio 1:25): C_p_—product reaction control (32 bp); C_S0_, C_S2_, C_S8_, C_S11_, C_S18_, C_S20_—DNA substrate control. The data were averaged from three independent experiments. The degree (%) of cleavage and the error are given in Table 1. Blue dots represent experimentally obtained values of substrate cleavage; green lines are the result of fitting by the theoretical model.

To compare the cleavage efficiency of all substrates under the same conditions, an excess of enzyme was used and the Cas9/sgRNA:dsDNA substrate ratio was 25:1. This ratio does not follow classical Michaelis–Menten kinetics; the amount of product quickly stabilizes at a level proportional to the molar ratio of Cas9/sgRNA to DNA [5]. Under these conditions, cleavage of the target substrate, a fully complementary sgRNA (S0 duplex), was approximately 40% with a half-reaction time of 3 min (Figure 1A and Table 1). To achieve a higher % cleavage, the Cas9/sgRNA:dsDNA substrate ratio should be increased [24]; therefore, the cleavage efficiency obtained for the fully complementary substrate was used as a reference against which the cleavage of mismatched substrates was evaluated. The substitutions were of the same type and did not affect the stability of the DNA duplexes and had approximately the same effect on the stability of the sgRNA/DNA complex. The difference in percentage of cleavage and the half-reaction time were primarily based on the position of the mismatched nucleotide pair. For most substrates (Table 1, Table S1 and Figure 1, Figures S2 and S3), the cleavage efficiency was lower and the half-time was longer. According to studies identifying the main factors determining the specificity of Cas9/RNA complex operation based on complementarity, the 6–8 bp located near the PAM fragment in the target DNA must perfectly match the sgRNA sequence, although the requirements become less strict when the enzyme is in abundance [10]. The lowest cleavage efficiency was observed for DNA duplex S8 (Figure 1D, cleavage percentage 22%, and half-time 18.5 min). Changing the mismatch G:dA to G:dG in the eighth position (S8gc) did not change the level of cleavage, and it remained as low as before; however, the half-reaction time increased by a factor of 2 (Table 1 and Figure S3A). This is consistent with the conclusion that the position of the guanidine G-pair forms quite strong base pairs with non-complementary bases, especially in the case of the G-G pair [12,25], which leads to an increase in reaction time. Low cleavage was also observed for substrate S13 (Table 1 and Figure S2), with a cleavage percentage of 29% and a half-reaction time of 15.5 min. This is consistent with the fact that the sensitivity to single nucleotide substitutions is maximal between 8 and 14 bp [26].

The exception was substrate S11 (Figure 1) with a higher percentage of cleavage (60%), but an almost fourfold longer half-reaction time (13 min) compared to substrate S0. Substitution at the 11th position with guanine results in the formation of a U:dG mismatch (S11gs) with a stronger interaction than for the U:dT pair (S11) [12,25], which leads to a decrease in the substrate cleavage to 42% and an increase in half-reaction time by almost a factor of 3 (S11gs, Table 1 and Figure S3B). Even so, the cleavage of the substrate S11gc remains higher than that for the complementary substrate S0.

Interesting results were obtained for substrates S18 and S20 (Table 1 and Figure 1). In the case of substrate S18, the cleavage and half-reaction time were comparable to S0, whereas the mismatch in substrate S20 resulted in a significantly higher cleavage (70%) with slightly less half-reaction time as for substrate S0. This phenomenon could be explained by the fact that the mismatch at the edge of the complementary sgRNA sequence site promotes substrate unfolding, facilitating the action of the enzyme. In both in vitro and in vivo studies, it has been shown that Cas9 is able to recognize and cleave DNA targets with mismatches that are located distal to the PAM sequence [26,27,28].

In the case of S20gc, changing the mismatch A:dA to A:dG leads to similar results as for S11gc: a decrease in the substrate cleavage to 53% and an increase in the half-reaction time by a factor of almost 10 (Table 1 and Figure S3C). Mismatches at distal positions are thought to cause a conformational change in Cas9 with activation of the nuclease domain [26,27]. The increase in the half-reaction time in the case of substates S8gc, S11gc, and S20gc could be explained by formation of the stronger interactions between the base pair in mismatches compared to substates S8, S11, and S20, which influence the initial duplex-unraveling processes due to a slightly higher stability, which requires more time for the cleavage reaction to occur.

A decrease in cleavage was observed for duplex S16 with the mismatch also located in the distal region. The efficiency of substrate cleavage was only 23%. Although substrates with mismatches up to and including 18 nucleotides relative to the PAM sequence are still well recognized and cleaved by the activated Cas9 complex, mismatches at the 17 or 16 positions lead to a significant reduction in enzyme efficiency (5–10-fold), while reducing substrate binding efficiency by only about 3-fold [28].

### 2.2. Thermodynamic Parameters of Cas9/sgRNA:dsDNA Complex Formation

To explain/understand the variation in cleavage efficiency observed for different positions of the mismatch in the sgRNA:DNA complex, and to confirm the hypothesis that substitutions have little impact on the stability of nucleic acid complexes, we performed molecular dynamics simulations of the binding of the most interesting substrates (S0, S2, S8, S8gc, S11, S11gc, S20, S20gc) to the Cas9/sgRNA complex. For the trajectories generated by the molecular dynamics simulations, we obtained thermodynamic parameters of duplex formation by analyzing the trajectories using the MM-GBSA method. Thermodynamic analysis of Cas9 complexes with nucleic acids was performed by separating the trajectory of the complex components from that of the initial system and calculating the binding energy of the complex components (Table 2).

We used substrates (dsDNA) as ligands and calculated the binding energy to the Cas9/sgRNA complex. For substrate S0, ΔE was −611.3 ± 3.0 kcal/mol. In the case of the mismatch at position 2 of the PAM fragment (S2), ΔE = −624.7 ± 3.0 kcal/mol. For substrates S8, S11, and S20, the value of ΔE was calculated to be −639.0 ± 3.1, −604.5 ± 2.9, and −592.3 ± 2.5 kcal/mol, respectively. For substrates with the G-C pair (S8gc, S11gc, and S20gc), the value of ΔE was also calculated (Table 2). Thus, all mismatches thermodynamically stabilize the formed sgRNA/DNA complex compared to the original DNA sequence (S0). Furthermore, the mismatch at position 2 from the PAM provides the greatest stabilization. The calculated data on the thermodynamic stability of sgRNA/DNA complexes do not correlate with the substrate cleavage data, supporting the hypothesis that the efficiency of Cas9 operation is more influenced by the co-localization of sgRNA/DNA and Cas9, as well as changes in the conformation of the enzyme, than by the thermodynamic stability of the sgRNA/DNA complex. Therefore, we decided to perform molecular dynamic modeling of the structure of a Cas9/sgRNA:dsDNA complex ready for cleavage. The complexes consisted of Cas9/sgRNA and dsDNA, so DNA was partially complexed with sgRNA in the so-called R-loop [26,29,30]. To calculate and compare the thermodynamic parameters of Cas9/sgRNA:dsDNA complexes, we analyzed the obtained 100 ns MD trajectories by molecular mechanics methods using the solvation energy of the continuum solvation (MM-GBSA).

All structures remain stable for 100 ns and the RMSD depending on the substrate is in the range of 5 Å when equilibrium is reached (Figure S4). A high stability of the Arg-binding site and the HNH domain is observed in all complexes. In substrate S2, the mismatching nucleotide is inverted from the duplex towards the Arg-binding domain compared to the original substrate S0. In addition, L II is shifted significantly closer to the protein surface relative to the duplex. For the S0 complex, L I and II are also labile, but the RMSD does not exceed 7 Å. The distances between the Mg^2+^ ions and the coordinating amino acid residues and water are conserved throughout the trajectory and mostly take the value of 2 Å.

In the case of duplexes with substitution for pairs of G-C, it is evident that the introduced substitutions also affect the mobility of the domains, mainly those that are already quite mobile, such as L, REC, and RuvC, but the C-terminal domain becomes slightly more fluctuating. There are no particular changes in the structure of the complex depending on the substrate context.

### 2.3. Structural Features of Cas9/sgRNA:dsDNA Complex Formation with Mismatches

**Figure 2:**
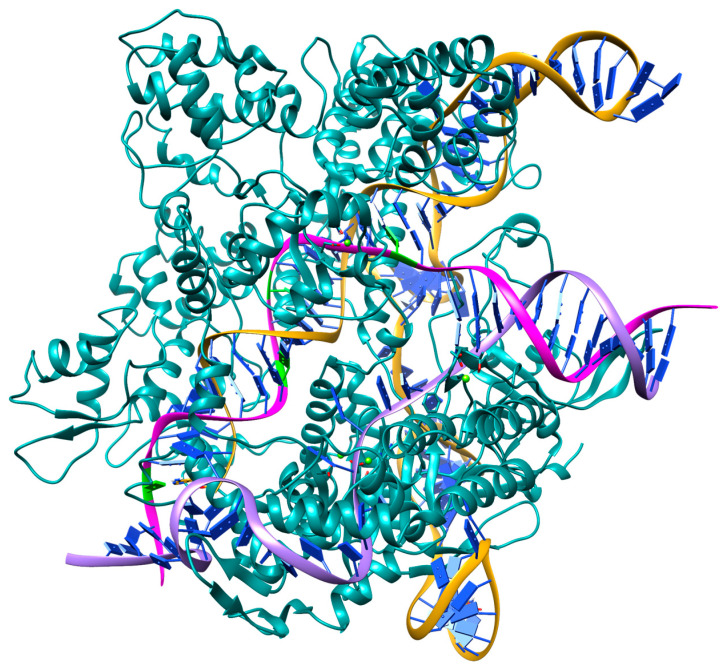
Three-dimensional structure of the Cas9/sgRNA:DNA (S0) complex. The sgRNA is shown in dark yellow; the DNA strand complementary to the sgRNA is shown in pink; the mismatches in positions are shown in green; and the second DNA strand is shown in purple.

Using MD simulations, the structures of Cas9/sgRNA:dsDNA complexes formed by interaction with the initial (S0), most (S20, S18, and S11), and least (S8) cleaved substrates were analyzed. Figure 2 shows the resulting structure of the Cas9/sgRNA complex with the S0 substrate, and for other substrates, the positions of mismatches in the chain complementary to the sgRNA are highlighted in green. The structure of the complex models an active system in which DNA is partially complexed with sgRNA in the R-loop [29,30].

Three mismatches in the PAM-distal region (positions 18–20) have been shown to activate Cas9 [18] through large conformational changes in the L1 and L2 linkers, resulting in the repositioning of the HNH domain to the target strand cleavage site.

Our experiments showed that the mismatch at the 20th position from the PAM sequence (S20) provides a more efficient activation, which results in a higher level of cleavage compared to the target sequence. At the same time, the S18 substrate is cleaved less efficiently due to the lack of coordination of the L1 linker with the linear form of the sgRNA:DNA in the initial stages of duplex formation. The efficient cleavage of the S11 substrate is due to the structural similarity of the Cas9/sgRNA complex with the Cas9/sgRNA complex with the S20 substrate, and the difference in cleavage time may be due to the shift in the L1 linker.

In the structures of the complexes, the HNH domain coordinates the target DNA strand and assumes a fully active conformation. The L1 helix forms a broad network of interactions with the sgRNA:DNA duplex in the PAM distal region. The L1 and L2 linkers were previously shown to maintain the HNH domain in the active conformation. The lack of coordination of the L1 linker with the linear form of the sgRNA:DNA duplex (in the initial stages of duplex formation) in the presence of mismatches in the PAM distal region does not prevent binding, but rapid cleavage does not occur in the case of some substrates. For example, a mismatch at position 18 does not decrease the percentage of cleavage, but it is 1.5 times slower.

The longest time with the lowest degree of cleavage was observed for substrate S8. In this case, the G:dA base pair is duplexed, and the structure of the sgRNA:DNA duplex is practically similar to that of the complex with substrate S0. Although the structure of the sgRNA:DNA duplex formed with S8 differs slightly from the original complex, the REC3 domain and the L1 linker are shifted closer to the nucleic acids. The amino acid Phe-916 is shown to be stacked with dA of the non-target chain, in contrast to the other structures.

The sgRNA:DNA duplex in the case of S11 contains an incorrect U:dT pair, which does not destabilize the duplex structure, and the base pairs are only slightly shifted relative to each other. The position of the Cas9 key domains involved in coordination and cleavage in this case is practically the same as in the interaction with S0. It is also interesting to note that the position of the dsDNA part of the structure is at a larger angle compared to the S0 substrate.

A comparison of the structure of the Cas9/sgRNA complex interacting with substrate S11 with the structures of the Cas9/sgRNA complexes interacting with S20 and S0 showed that the position of helix L1 is slightly different in the case of substrate S11, but this is in synchrony with the shift in the duplex slightly inside the protein. The position of the REC3 domain in the S11 and S20 complexes is practically not different at the end of the MD simulation, while there is a noticeable difference with its position in S0. It is likely that the high similarity of the structures after 500 ns of MD can explain the high percentage of substrate cleavage, and the longer reaction time in the case of the S11 substrate may be determined by the L1 shift.

## 3. Materials and Methods

### 3.1. Materials

All chemical compounds used in this work—Tris, HEPES dithiothreitol (DTT), EDTA, NaCl, KCl, MgCl_2_, Imidazol, glycerol, and others—were acquired from Sigma (St. Louis, MO, USA) or Molekula GmbH (München, Germany). The plasmid encoding a Cas9 from S. pyogenes (spyCas9), containing 6x-Histidine-tagged protein and a TEV site (pMJ806), was obtained from Addgene (Watertown, MA, USA). HiTrap Chelating HP and Heparine HP columns were purchased from GE Healthcare (GE 266 Healthcare, Chicago, IL, USA). TEV protease and T7 RNA polymerase proteins were purchased from Biolabmix (Novosibirsk, Russia). Kanamycin sulfate was acquired from Fluorochem (Hadfield, UK). Isopropyl-*β*-D-1-thiogalactopyranoside (IPTG) was provided by Thermo Fisher Scientific (Abingdon, UK).

### 3.2. Cas9 Protein

The Cas9 protein was obtained similarly to the protocol described in [31,32] with some modifications. In the first step, the plasmid encoding a Cas9 was transformed into *E. coli* BL21 cells according to a standard heat shock protocol, as described in [33]. Then, the protein was expressed in *E. coli* BL21 by growing in Luria Broth medium supplemented with Kanamycin (50 µg/mL). Protein expression was induced by adding isopropyl-β-D-1-thiogalactopyranoside to a final concentration of 1 mM and growing for 18 h at 18 °C. Cells were lysed using a French Press (Glen Mills, Inc., Clifton, NJ, USA) at 40,000 psi of pressure. The clarified lysate was bound to a HiTrap Chelating HP column (5 mL, GE 266 Healthcare, Chicago, IL, USA). The column was charged with nickel (Ni^2+^) ions before use. The recombinant (histidine)6-tagged Cas9 was eluted by a line gradient of imidazole in buffer (20 mM Tris-HCl, pH 8.0, 300 mM NaCl, 250 mM Imidazol). Further, 0.5 mg of TEV protease was added per 50 mg of protein, and the sample was dialyzed in dialysis tubing (Scienova GmbH, Jena, Germany) with a molecular weight cut-off of 12–14 kDa against the buffer (20 mM HEPES-KOH, pH 7.6, 150 mM KCl, 10% (*v*/*v*) glycerol, 1 mM dithiothreitol (DTT), 1 mM EDTA) at 4 °C overnight.

The Cas9 protein eluent was additionally purified on a Heparine HP column (GE Healthcare, Chicago, IL, USA). Bound protein was eluted in a line gradient of KCl in two steps (30 and 50%) in buffer (20 mM HEPES-KOH, pH 7.6, 1 M KCl, 10% glycerol (*v*/*v*), 1 mM DTT). The concentration of the purified enzyme was determined by UV absorbance at 280 nm using the NanoDrop OneC device (Thermo Scientific, Medison, WI, USA).

### 3.3. sgRNA

The sgRNA with the following sequence, 5′-pppGGAUAACUCAAUUUGUAAAAAAGUUUUAGAGCUAGAAAUAGCAAGUUAAAAUAAGGCUAGUCCGUUAUCAACUUGAAAAAGUGGCACCGAGUCGGUGCUUUU-3′), was prepared by in vitro transcription by T7 RNA polymerase and a DNA matrix as described in [32]. The transcription template was obtained by amplification from the plasmid MLM3636T7promSp2 (#43860, Addgene, Watertown, MA, USA). The reaction mixture (300 µL) for sgRNA production consisted of phage T7 RNA polymerase buffer (40 mM Tris-HCl (pH 8.0), 2 mM spermidine, 8 mM MgCl_2_, 25 mM NaCl), 0.4 mM NTP (Biosan, Novosibirsk, Russia), 5 mM DTT, 5 nM DNA matrix, and 3 µM (1 U/μL) phage T7 RNA polymerase. The reaction mixture was incubated at 37 °C for 1 h in ThermoStat plus (Eppendorf, Hamburg, Germany). After in vitro transcription, DNAase I (Thermo Scientific, Medison, WI, USA) was added at a concentration of 1.5 U/μL and incubated at 37 °C for 15 min in ThermoStat plus (Eppendorf, Hamburg, Germany). Then, sgRNA was precipitated from the reaction mixture by adding 900 µL of 96% EtOH and 30 µL of 3 M NaOAc (pH 5.2) and incubated at −20 °C for 1 h followed by centrifugation at 15,500× *g* for 15 min at 4 °C. The supernatant was removed and the precipitate was washed twice with 70% EtOH cooled to −20 °C. The precipitate was dried in a CentriVap concentrator (Labconco, Kansas City, MO, USA) at 24 °C. The sgRNA obtained was further purified in 8% polyacrylamide gel containing 8 M urea. The sgRNA was eluted from the gel by two volumes of buffer (1 mM EDTA-KOH (pH 8.0), 500 mM NH4OAc) for 3 h at 25 °C. Subsequently, the obtained solution was filtered on Ultrafree-CL columns (Merck Millipore, Burlington, MA, USA). The sgRNA was precipitated in a similar manner as described above and stored dry at −70 °C. The concentration was measured using a NanoDrop One spectrophotometer (Thermo Scientific, Medison, WI, USA).

### 3.4. Oligonucleotide Substrates

All oligonucleotides, used in this study as substrates (Table 1), were synthesized at the Laboratory of Synthetic Biology of the ICBFM SB RAS on ASM-800 or ASM-2000 DNA/RNA synthesizers (BIOSSET, Novosibirsk, Russia) using the standard phosphoramidite method. The 6-FAM phosphoramidites (Lumiprobe, Moscow, Russia) were used to synthesize the fluorescently labeled oligonucleotides. The products were purified in 12% PAAG with subsequent desalting on homemade cartridges containing C18 reverse-phase column (Waters, Milford, MA, USA).

The resulting oligonucleotides were characterized on an Orbitrap Q Exactive HF high-resolution mass spectrometer (Thermo Scientific, Inc., Waltham, MA, USA) in the 1900–3500 Da range at the Core Facility of Mass Spectrometric Analysis of the Institute of Chemical Biology and Fundamental Medicine SB RAS (Table S1).

To form the DNA substrates (S1–S20, Table 1), equimolar amounts of complementary single-strand oligonucleotides were incubated for 5 min at 95 °C in buffer consisting of 10 mM Tris/HCl, pH 8, and 50 mM NaCl with a total volume of 50 µL. The samples were then slowly cooled to room temperature. The resulting ds oligonucleotide was stored at −20 °C.

### 3.5. Reactions with Oligonucleotide Substrates

To activate the enzyme, Cas9 (100 nM) was pre-incubated with sgRNA (100 nM) at an equimolar ratio for 15 min at 37 °C in water, thereby forming the complex Cas9/sgRNA. The volume of the reaction mixture is 90 μL.

The cleavage reaction was initiated by adding ds oligonucleotide (to a final concentration of 2 nM) to the activated Cas9/sgRNA complex. An amount of 160 μL of the reaction mixture was incubated at 37 °C in buffer (10 mM Tris-HCl, pH 7.5, 100 mM NaCl, 1 mM DTT, 10 mM MgCl_2_). At different time points (from 2.5 to 270 min), 20 µL of the reaction mixture was transferred to new tube, and the reaction was stopped by adding 10 μL of a solution containing 90% formamide, 50 mM EDTA, 0.01% bromophenol blue, and 0.01% xylene cyanol, with incubation at 95 °C for 5 min. The reaction products were analyzed by electrophoresis in 15% polyacrylamide gel containing 7 M urea, followed by gel scanning on a VersaDoc (BioRad, Hercules, CA, USA).

### 3.6. Determination of Parameters

Gels were processed in Gel-Pro Analyzer v.4 software. The intensity of each band was turned into numbers and the time dependence of cleavage accumulation was plotted. The yield of cleavage was calculated from the kinetic data using exponential fitting with the OriginPro 2015 software and is represented as an exponential equation curve, y=A1×exp(−xt1)+y0, in which *y* represents the percentage of cleaved substrate, A1 is amplitude, and *x* is reaction time. The errors of the values were within 10–30%.

### 3.7. Computer Simulation

#### 3.7.1. Molecular Dynamics Simulation

A three-dimensional structure with PDB ID 7S4X [30] was used as the starting structure of the protein. Using UCSF Chimera 1.16 [34], the missing regions of the protein structure were completed and the necessary nucleic acid sequences were obtained. The complexes consisted of Cas9 with sgRNA and dsDNA, so the DNA was partially in complex with the sgRNA, in a so-called R-loop [29]. The model complexes contained Cas9 (1364 a.o.), sgRNA (98 nucleotides), and dsDNA (36 bp) and Mg^2+^ ions in the active centers. The lengths of the nucleic acids were reduced relative to the experimental ones to reduce the fluctuation in their ends during the molecular dynamics simulation.

The Amber20 [35] ff19SB force field [36] was used for protein, OL3 was used for sgRNA molecules, bsc1 was used for DNA [37], and the TIP3P force field was used for water, metal ions, and counterions [38]. Prior to molecular dynamics simulations, the energies of the systems were minimized in an implicit solvent model using the sander module of the Amber20 program. After pretreatment, the systems were neutralized with Na^+^ ions and solvated with water molecules using tLEaP 4.2.0. TIP3P with truncated “cubic” periodic boundary conditions with an 8 Å spacing was used as the explicit water model.

The energy minimization was performed in two steps. After the energy minimization step, each system containing Cas9 was gradually heated from 1 to 298 K for 1 ns with periodic boundary conditions. Equilibrium molecular dynamics simulations of 100 ns in duration for Cas9 and their complexes with nucleic acids were performed in the NPT ensemble. The resulting trajectories were analyzed using UCSF Chimera 1.16 and CPPTRAJ 2.1.0 [39] programs.

#### 3.7.2. MM-GBSA Calculations

We analyzed the obtained molecular dynamics trajectories using molecular mechanics energies combined with the molecular mechanics energies combined with the generalized Born and surface area continuum solvation (MM-GBSA) to calculate the thermodynamic parameters of the Cas9/RNA:dsDNA complexes.

We generated the topology files for the components of complexes being studied prior to analysis using CPPTRAJ 2.1.0. The ionic strength of the solution was assumed to be 100 mM monovalent cations. In the calculations, each step of the MD trajectory was used.

## 4. Conclusions

Our data confirm that substitutions in the target DNA by non-complementary nucleotides to the sgRNA do not result in the absence of substrate cleavage by the Cas9 enzyme, although they significantly reduce the catalytic activity of the Cas9/sgRNA complex, depending on the location of the mismatch. Activation of the Cas9/sgRNA complex is demonstrated for substrates with substitutions at positions 11, 18, and 20 relative to PAM, although the stability of all DNA duplexes differs only slightly. Molecular modeling data show that the efficiency and depth of the Cas9 cleavage reaction are not determined by the thermodynamic parameters of the system, but by the topology of the key domains involved in substrate recognition and cleavage. The position of the mismatch in the substrate is also an additional influencing factor.

The efficiency of Cas9 operation is mainly affected by the conformational changes in Cas9 and the mutual arrangement of sgRNA and substrates.

We believe that the data presented (as part of a large body of such data) will allow for further targeting to influence the efficiency and targeting of Cas9-mediated DNA cleavage.

## Figures and Tables

**Table 1:** Efficiency of cleavage of 55 bp DNA substrates containing a mismatch in the “seed” sequence.

Name	Sequence (5′→3′ Complementary RNA)	% Cleavage	t_1/2_, min
S8gc	GCACTGCAGGAACTCTACCA**TTT′TTTAGAAATTGAGTTAT**TAAGAGGGGGGGTCC CGTGACGTCCTTGAGATGGTAAA′AAAT**C**TTTAACTCAATAATTCTCCCCCCCAGG	19.0 ± 0.7	39.7 ± 5.8
S8	GCACTGCAGGAACTCTACCA**TTT′TTTAAAAATTGAGTTAT**TAAGAGGGGGGGTCC CGTGACGTCCTTGAGATGGTAAA′AAATTTTTAACTCAATAATTCTCCCCCCCAGG	21.9 ± 1.0	18.5 ± 5.7
S16	GCACTGCAGGAACTCTACCA**TTT′TTTACAAATTGAATTAT**TAAGAGGGGGGGTCC CGTGACGTCCTTGAGATGGTAAA′AAATGTTTAACTTAATAATTCTCCCCCCCAGG	22.6 ± 1.1	5.7 ± 1.2
S6	GCACTGCAGGAACTCTACCA**TTT′TTAACAAATTGAGTTAT**TAAGAGGGGGGGTCC CGTGACGTCCTTGAGATGGTAAA′AATTGTTTAACTCAATAATTCTCCCCCCCAGG	23.0 ± 1.4	9.8 ± 2.7
S13	GCACTGCAGGAACTCTACCA**TTT′TTTACAAATAGAGTTAT**TAAGAGGGGGGGTCC CGTGACGTCCTTGAGATGGTAAA′AAATGTTTATCTCAATAATTCTCCCCCCCAGG	28.9 ± 1.2	15.5 ± 2.3
S2	GCACTGCAGGAACTCTACCA**TAT′TTTACAAATTGAGTTAT**TAAGAGGGGGGGTCC CGTGACGTCCTTGAGATGGTATA′AAATGTTTAACTCAATAATTCTCCCCCCCAGG	31.0 ± 1.1	13.5 ± 2.1
S4	GCACTGCAGGAACTCTACCA**TTT′ATTACAAATTGAGTTAT**TAAGAGGGGGGGTCC CGTGACGTCCTTGAGATGGTAAA′TAATGTTTAACTCAATAATTCTCCCCCCCAGG	35.8 ± 1.2	5.2 ± 0.9
S5	GCACTGCAGGAACTCTACCA**TTT′TATACAAATTGAGTTAT**TAAGAGGGGGGGTCC CGTGACGTCCTTGAGATGGTAAA′ATATGTTTAACTCAATAATTCTCCCCCCCAGG	36.9 ± 2.4	13.2 ± 2.4
S0	GCACTGCAGGAACTCTACCA**TTT′TTTACAAATTGAGTTAT**TAAGAGGGGGGGTCC CGTGACGTCCTTGAGATGGTAAA′AAATGTTTAACTCAATAATTCTCCCCCCCAGG	38.0 ± 0.8	4.8 ± 0.7
S18	GCACTGCAGGAACTCTACCA**TTT′TTTACAAATTGAGTAAT**TAAGAGGGGGGGTCC CGTGACGTCCTTGAGATGGTAAA′AAATGTTTAACTCATTAATTCTCCCCCCCAGG	39.0 ± 3.6	4.5 ± 1.9
S11gc	GCACTGCAGGAACTCTACCA**TTT′TTTACAAGTTGAGTTAT**TAAGAGGGGGGGTCC CGTGACGTCCTTGAGATGGTAAA′AAATGTT**C**AACTCAATAATTCTCCCCCCCAGG	42.5 ± 0.6	32.2 ± 1.3
S20gc	GCACTGCAGGAACTCTACCA**TTT′TTTACAAATTGAGTTAG**TAAGAGGGGGGGTCC CGTGACGTCCTTGAGATGGTAAA′AAATGTTTAACTCAAT**C**ATTCTCCCCCCCAGG	53.4 ± 0.8	25.3 ± 1.1
S11	GCACTGCAGGAACTCTACCA**TTT′TTTACAATTTGAGTTAT**TAAGAGGGGGGGTCC CGTGACGTCCTTGAGATGGTAAA′AAATGTTAAACTCAATAATTCTCCCCCCCAGG	60.4 ± 7.8	12.8 ± 4.3
S20	GCACTGCAGGAACTCTACCA**TTT′TTTACAAATTGAGTTAA**TAAGAGGGGGGGTCC CGTGACGTCCTTGAGATGGTAAA′AAATGTTTAACTCAATTATTCTCCCCCCCAGG	69.0 ± 1.8	2.6 ± 0.4

DNA substrates consist of completely complementary strands but form an incorrect complex with sgRNA. The cleavage positions are shown as ′. Sequence complementary to sgRNA is shown in bold. GGT—PAM; t_1/2_—half-time of DNA substrate cleavage.

**Table 2:** Values of complex formation energies obtained with MM-GBSA.

Complex	Receptor	Ligand	ΔE, kcal/mol
Cas9/sgRNA:dsDNA (S0)	Cas9/sgRNA	dsDNA (S0)	−611.3 ± 3.0
Cas9/sgRNA:dsDNA (S2)	Cas9/sgRNA	dsDNA (S2)	−624.7 ± 3.0
Cas9/sgRNA:dsDNA (S8)	Cas9/sgRNA	dsDNA (S8)	−639.0 ± 3.1
Cas9/sgRNA:dsDNA (S8gc)	Cas9/sgRNA	dsDNA (S8gc)	−542.5 ± 3.6
Cas9/sgRNA:dsDNA (S11)	Cas9/sgRNA	dsDNA (S11)	−604.5 ± 2.9
Cas9/sgRNA:dsDNA (S11gc)	Cas9/sgRNA	dsDNA (S11gc)	−585.6 ± 2.8
Cas9/sgRNA:dsDNA (S20)	Cas9/sgRNA	dsDNA (S20)	−592.3 ± 2.5
Cas9/sgRNA:dsDNA (S20gc)	Cas9/sgRNA	dsDNA (S20gc)	−541.8 ± 3.1

## Data Availability

Data are available on request, owing to privacy and ethical restrictions.

## Supplementary Materials

The following supporting information can be downloaded at https://www.mdpi.com/article/10.3390/ijms251910862/s1.
