# Supplementary material for: Cleavage of DNA Substrate Containing Nucleotide Mismatch in the Complementary Region to sgRNA by Cas9 Endonuclease: Thermodynamic and Structural Features
Source: Int J Mol Sci. 2024 Oct 9;25(19):10862. doi: 10.3390/ijms251910862 (PMC11476762; doi:10.3390/ijms251910862)
Supplement: Supplementary file 1 [file ijms-25-10862-s001.zip › ijms-3158770-supplementary.pdf]

Supplementary Materials

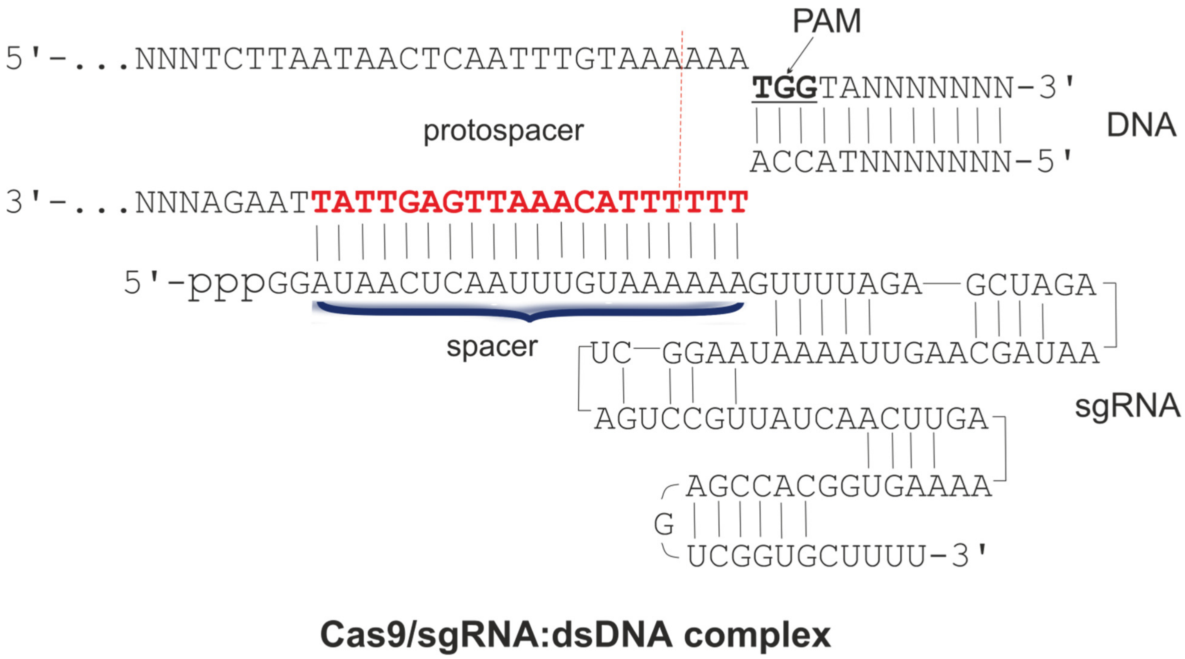

Figure S1. Illustration of the complex between Cas9-sgRNA and a DNA substrate.

Table S1. Sequences and masses of oligonucleotides

№	Name	Sequence Oligonucleotide (5'→3')	Theoretical (Average) Mass M, Da	Measured M, Da
1	S0	GCACTGCAGGAACTCTACCATTTTTTACAAATTGAGTTATTAAGAGGGGGGGTCC	17003,98	17529,75 (+23Na)
2	S2	GCACTGCAGGAACTCTACCAT**A**TTTTACAAATTGAGTTATTAAGAGGGGGGGTCC	17012,99	17425,06 (+18Na)
3	S4	GCACTGCAGGAACTCTACCATTT**A**TTACAAATTGAGTTATTAAGAGGGGGGGTCC	17012,99	17403,07 (+17Na)
4	S5	GCACTGCAGGAACTCTACCATTTT**A**TACAAATTGAGTTATTAAGAGGGGGGGTCC	17012,99	17319,82 (+13Na)
5	S6	GCACTGCAGGAACTCTACCATTTTT**A**ACAAATTGAGTTATTAAGAGGGGGGGTCC	17012,99	17495,79 (+21Na)
6	S8	GCACTGCAGGAACTCTACCATTTTTTA**A**AAATTGAGTTATTAAGAGGGGGGGTCC	17028	17027,94
7	S11	GCACTGCAGGAACTCTACCATTTTTTACAA**T**TTGAGTTATTAAGAGGGGGGGTCC	16994,97	16994,97
8	S13	GCACTGCAGGAACTCTACCATTTTTTACAAAT**A**GAGTTATTAAGAGGGGGGGTCC	17012,99	17012,91
9	S16	GCACTGCAGGAACTCTACCATTTTTTACAAATTGA**A**TTATTAAGAGGGGGGGTCC	16987,98	17033,75 (+2Na)
11	S18	GCACTGCAGGAACTCTACCATTTTTTACAAATTGAGT**A**ATTAAGAGGGGGGGTCC	17012,99	17012,92
12	S20	GCACTGCAGGAACTCTACCATTTTTTACAAATTGAGTTA**A**TAAGAGGGGGGGTCC	17012,99	17219,65 (+9Na)
13	S8gc	GCACTGCAGGAACTCTACCATTTTTTAGAAATTGAGTTATTAAGAGGGGGGGTCC	17044,15	17043,09
14	S11gc	GCACTGCAGGAACTCTACCATTTTTTACAAGTTGAGTTATTAAGAGGGGGGGTCC	17020,13	1719,25
15	S20gc	GCACTGCAGGAACTCTACCATTTTTTACAAATTGAGTTAGTAAGAGGGGGGGTCC	17029,14	1731,91

Oligonucleotides were analyzed on an Orbitrap Q Exactive HF high-resolution mass spectrometer (Thermo Scientific, Inc., Waltham, MA, USA) in the 1900-3500 Da range. In this case multiply charged ions were detected (4^+, 5^+, 6^+, 7^+ and 14^+). The deconvolution of oligonucleotide spectra was carried out using the BioPharma Finder™ (v 5.2, Thermo Fischer Scientific, Waltham, MA, USA). In some cases, oligonucleotides were detected as a Na^+ adducts containing between one and 23 sodium ions.

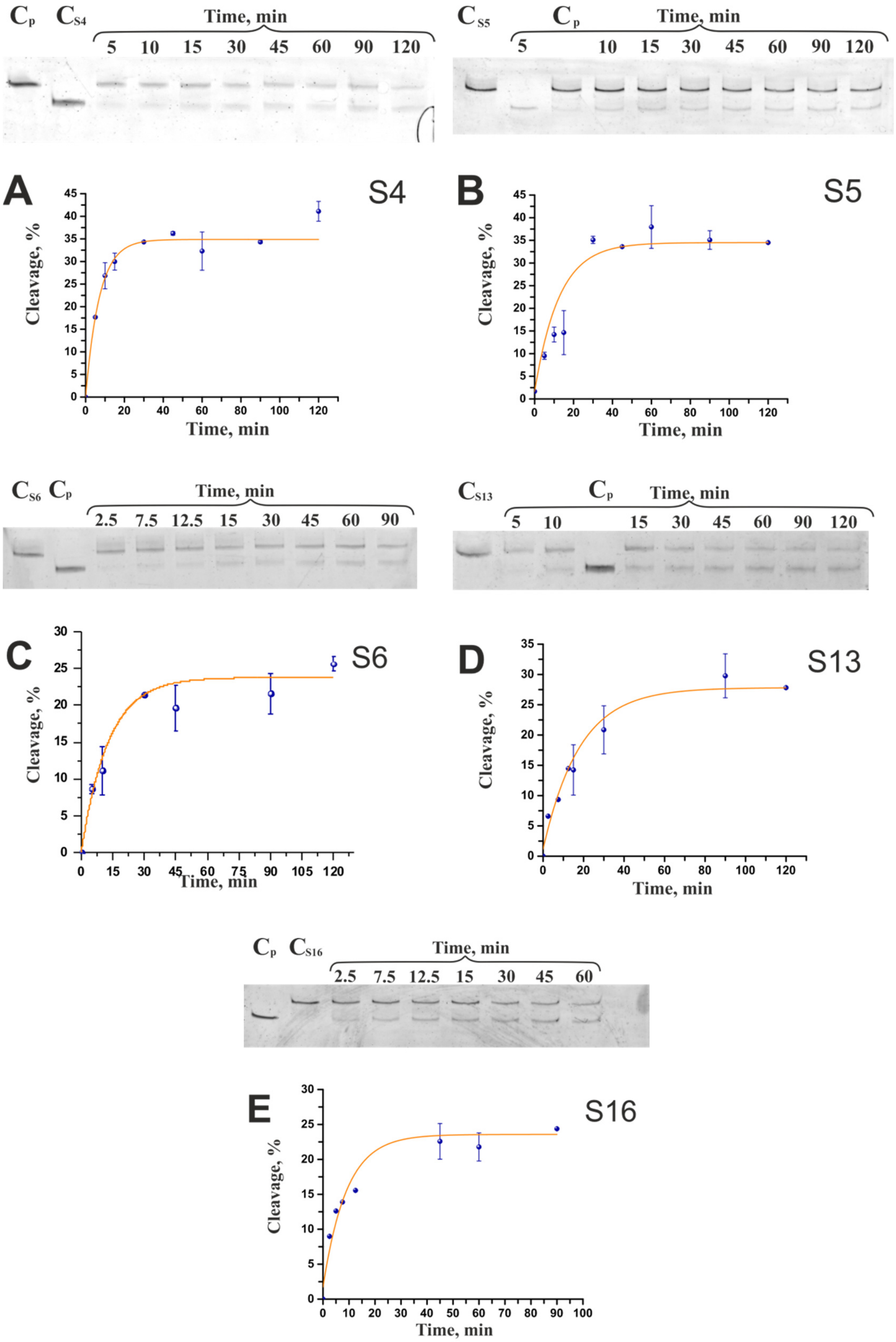

Figure S2. Cleavage assay of 55 b.p. substrate S4 (**A**), S5 (**B**), S6 (**C**), S13 (**D**) and S16 (**E**) analyzed by denaturing 15% polyacrylamide gel electrophoresis and representation of time dependence of cleavage on graphs. Cleavage was performed using 2 nM FAM-labelled dsDNA and 50 nM complex Cas9/sgRNA (ratio 1:25): C_p - product reaction control (32 b.p.), C_{S4}, C_{S5}, C_{S6}, C_{S13}, C_{S16}-DNA substrate control. The data were averaged from three independent experiments. The degree (%) of cleavage and the error are given in Table 1.

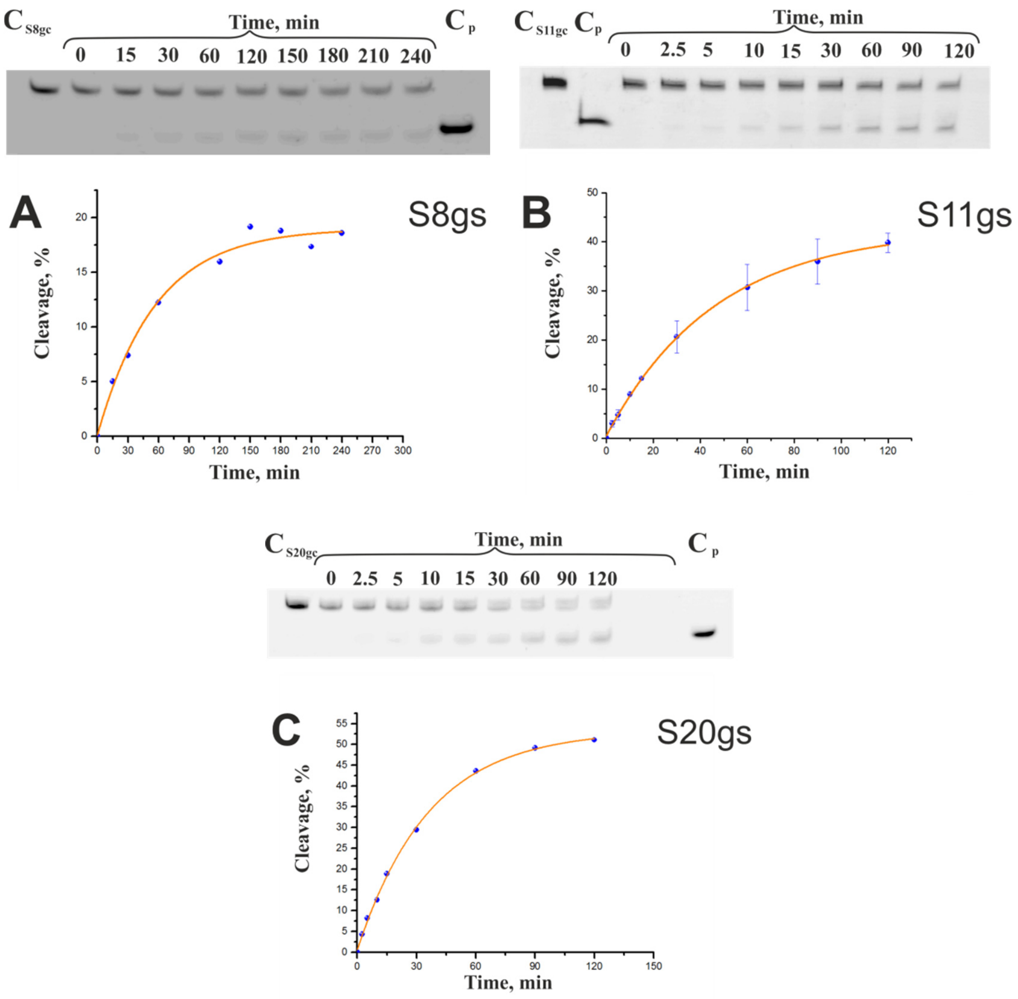

Figure S3. Cleavage assay of 55 b.p. substrate S8gc (**A**), S11gc (**B**) and S20gc (**C**) analyzed by denaturing 15% polyacrylamide gel electrophoresis and representation of time dependence of cleavage on graphs. Cleavage was performed using 2 nM FAM-labelled dsDNA and 50 nM complex Cas9/sgRNA (ratio 1:25): C_P - product reaction control (32 b.p.), C_{S8gc}, C_{S11gc}, C_{S20gc} - DNA substrate control. The data were averaged from three independent experiments. The degree (%) of cleavage and the error are given in Table 1.

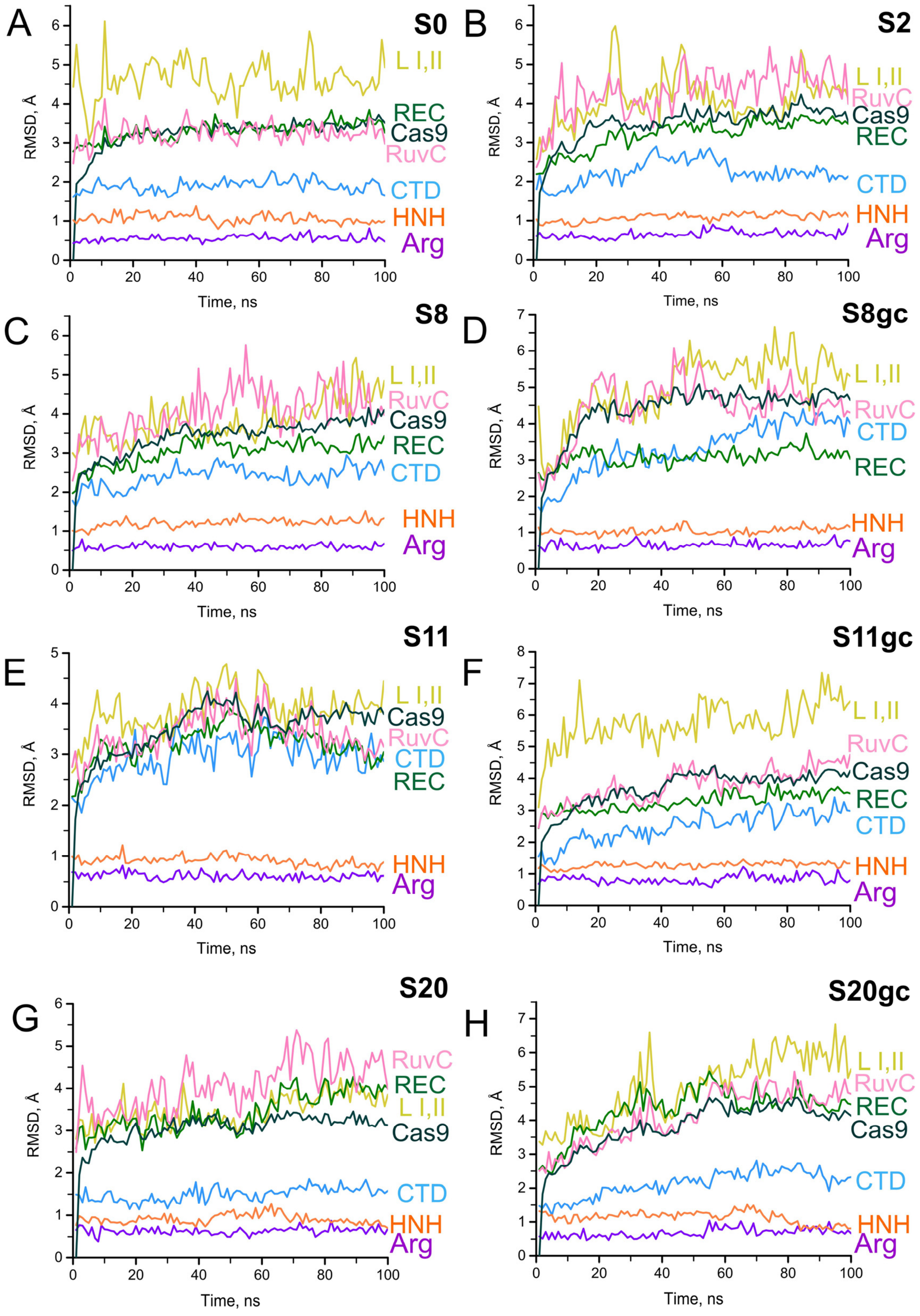

Figure S4. Time evolution of RMSD values (in Angstroms, Y-axis) calculated from 100 ns trajectories (X-axis) for Cas9/sgRNA complexes with dsDNA S0 (**A**), S2 (**B**), S8 (**C**), S8gc (**D**), S11 (**E**) S11gc (**F**), S20 (**G**) and S20gc (**H**). Average RMSD values for protein (darck green), REC (green), CTD (blue), RuvC (pink) and HNH (orange) domains, L-I-II linkers (yellow) and Arg (purple) are shown. The amplitude of the deviation correlates with the dynamic behaviour of the protein and its domains.
